# Accelerating access to medicines in a changing world

**DOI:** 10.2471/BLT.19.249664

**Published:** 2020-07-08

**Authors:** Hannah Kettler, Susanna Lehtimaki, Nina Schwalbe

**Affiliations:** a Seattle, United States of America (USA).; b Spark Street Advisors, New York, USA.; c Heilbrunn Department of Population and Family Health, Mailman School of Public Health, Columbia University, 722 West 168^th^ Street, New York, 10032, USA.

Improving access to quality medicines is essential to achieve the sustainable development goals and universal health coverage.^1^ Over the past two decades, the global health community has invested to accelerate access to medicines for the world’s poorest people through increasingly sophisticated mechanisms. Fuelled by new public, private and philanthropic resources, these investments have yielded important results.^2^ However, with poverty projected to increase, including due to the coronavirus disease 2019 (COVID-19) and fewer countries eligible for aid (as they become middle-income countries),^3^ a stronger link between what patients and countries need and can pay for, and what medicines donors and companies develop and supply, will be particularly important in optimizing access to medicines.

## Access tools and approaches

Challenges in access to medicines include affordability, availability, acceptability and accessibility. Over the past two decades, multiple access tools have been developed to address each of these challenges, with a focus on diseases that disproportionately affect people living in low-income countries. To understand the context and range of access tools being deployed and to offer suggestions for moving forward, we surveyed changes in the health landscape, reviewed the access efforts of the Bill & Melinda Gates Foundation (BMGF) and identified over 40 tools (Box 1). These tools include both supply- and demand-side interventions, pooled procurement to volume guarantees, patent pools, research and development partnerships, advance market commitments and product and manufacturing innovations.

Box 1 **Access tools addressing the four challenges of access to medicines**

**Improve affordability**

Global access plans; product development partnerships; cost–efficient manufacturing platforms; test with fewer or lower vaccine doses; reduce active pharmaceutical ingredient; promote voluntary licensing; employ patent pool; and negotiate lower prices for lower-income countries (through tiered pricing, advanced market commitment, pooled procurement, price subsidy, volume guarantee, buy downs, advanced market commitment, pooled procurement, price subsidy and volume guarantee).

**Improve accessibility**

Cold chain innovation; improve logistics; strategic purchasing to improve supply chains; technology- enabled delivery; behaviour economics; primary health-care performance initiative; and data systems, surveillance and analysis.

**Increase availability**

Humanitarian license; technical assistance; policy incentives; expand clinical trials network; translational research design; WHO prequalification system strengthening; policy guidance process; pharmacovigilance; regional regulatory harmonization; supply chain support; demand forecasts; and accelerated development and introduction plans /market manager.

**Improve acceptability**

Research and development based on patient need informed target product profiles; user-centred design; precision public health campaigns; surveillance, disease burden studies; health technology assessment capacity building; discovery accelerator; grand challenge explorations; improve products: fewer injections, appropriate packaging, thermostability, reduce overage; and invest in improved adherence technologies and approaches.

These tools primarily target communicable diseases and include both global diseases and diseases of poverty. Global diseases, including those caused by human immunodeficiency virus (HIV), pneumococcus and rotavirus, are prevalent in both high- and low-income countries. Diseases of poverty, including malaria, tuberculosis and lymphatic filariasis are mostly found in low-income countries, and increasingly in the poorest population segments in middle-income countries.

For rotavirus, companies brought vaccines into the market in response to demand from high-income countries (Box 2). Access tools have focused on creating demand and on vaccine pricing and supply. Following early investment through the Accelerated Development and Introduction Plan Initiative, Gavi, the vaccine alliance, offered companies volume guarantees in exchange for supply at the lowest tier pricing. The Gates Foundation and others funded research to reduce product cost, and provided technical assistance on manufacturing and production and regulatory issues to help local companies enter the market. As of 2019, four products had obtained the World Health Organization’s prequalification. Despite previous product shortages, supply currently meets demand.

Box 2 **Case examples of access tools of medicine: rotavirus vaccine and tuberculosis therapies**

**Access Tools for Global Diseases – Rotavirus Vaccine**

1. Generate

a) Demand aggregation and pooled procurement (for instance through Gavi, the Vaccine Alliance)

b) Volume guarantees

c) Demand creation (for example Rota Advanced Development and Introduction Partnership)

2. Support competition

a) Grants and technical assistance to support additional suppliers

b) Build enabling systems, such as the World Health Organization’s, for example its prequalification systems, policy/norms and standards

3. Innovate to improve originator product

a) Manufacturing platforms to bring down costs

b) Innovation in dosing, packaging, thermostability/cold chain

**Access tools for diseases of poverty – tuberculosis therapies**

1. Manage intellectual property for future global access

a) Global access plans with manufacturers

b) Non-exclusive license via a humanitarian license clause

2. De-risk product development

a) Financing for product development and dedicated translational research

b) Development and advocacy for policy incentives

3. Accelerate product pathways

a) Support for development of accelerated regulatory and clinical trials pathways and capacity in high income and target countries

b) Support use of real-world evidence to improve clinical trial design

4. Accelerate product uptake

a) Country prequalification

b) Supply chain readiness

c) Finance for product introduction

d) Country decision-making including health technology assessment capacity decision-making

5. Align product design with user needs

a) Adherence to target product profiles

b) Improved formulations and product design

c) Platform technologies to improve adherence, delivery

Pneumococcal disease is another example of a global disease, where the pilot advance market commitment encouraged the scale up of production of affordable vaccines tailored to the needs of low-income countries. As of 2019, 60 Gavi-eligible countries had introduced the vaccine through this mechanism.

For diseases of poverty, a multicompany, early-stage, public-private research initiative, expedited regulatory pathways, push funding and target product profiles are signature components of an access tools portfolio. For tuberculosis, for example, which lacks strong market incentives, access tools have focused on accelerating the development and introduction of appropriate and affordable products, including new combination therapies, vaccines and diagnostics. 

As of 2020, while corporate and non-profit sponsors have moved new regimens into clinical development, financing for late-stage development and product introduction and uptake strategies is still needed. By contrast, for malaria, access efforts have included both the demand-side with the affordable medicines facility and supply-side interventions. 

## Global health landscape and access

Several demographic and economic shifts, likely to be exacerbated by COVID-19, are important to consider. First, while access initiatives traditionally focused on low-income countries, a higher number of poor people live in middle-income countries.^4^ Of the 700 million people living in extreme poverty as of 2019, most lived in middle-income countries such as India, Indonesia and Nigeria, countries that were expected to transition away from development aid-eligibility.^4^ By 2019, global poverty had dropped rapidly at the individual and aggregate levels, and the proportion of countries classified as middle- or high-income grew from 70% in 2000 (144/205) to 86% in 2015 (187/218).^5^ However, many of these countries demonstrate significant in-country disparities in terms of morbidity, mortality and average per capita income levels.^6^

Second, a growing proportion of low-income and aid-eligible countries are either fragile or volatile.^7^ Poor access to medicines in these countries is a consequence of a lack of trained providers or weak health-care delivery systems. Also, in countries with migrant populations, reaching populations on the move may require tailoring product characteristics to people who are far from health-care delivery points and who are not able to store their own medicines.

Third, the products required to meet the needs of the poorest are shifting. Many low- and middle-income countries face an epidemiological transition, with disease burden shifting from infections to noncommunicable diseases, such as cardiovascular disease, cancer and diabetes.^8^ As such, they also need to procure new products for these diseases, many of which have never been covered by concessional pricing schemes. Middle-income countries, where dual public and private sector procurement channels exist, will likely require differentiated access strategies.^9^

Finally, in both low- and middle-income countries, domestic health financing has been increasing in absolute terms and relative to other sources, and is expected to further increase over time, with households bearing the substantial share of the costs. In low- and lower-middle-income countries, out-of-pocket expenses constitute between 42–56% of total health expenditures.^10^ Government resources cover between 26–32%, while development assistance for health represents 25% of total health expenditures in low-income countries, and only 3% in lower middle-income countries.^10^

These shifts have significant implications for access initiatives. Global access plans, global procurement funds, inter-country tiered pricing and voluntary licenses agreements granted for specific countries, were all developed for a world where most beneficiaries were concentrated in aid-eligible low-income countries. Companies were prepared to offer concessionary pricing in exchange for high volumes and a single buyer to reduce transaction costs, such as Gavi and the United Nations Children’s Fund. With fewer of the poorest people living in aid-eligible countries, securing volume-based price reductions and aid-subsidized tiered pricing from pharmaceutical companies will be harder. Also, the potential for sales to private markets as income grows may deter companies from agreeing to country-wide pricing agreements. To reach the poorest people, differentiating access provisions within (and not just between) countries will be necessary.

Since many countries that remain aid-eligible are fragile or politically volatile, demand is uncertain, exacerbating the problem of negotiating sustained low-price supply contracts. Even where prices have been negotiated by or on behalf of the government, services are often provided and paid for by non-state actors, such as humanitarian organizations, who may not have access to these pricing discounts.

Furthermore, the dual disease burden requires governments to secure access for treatments for patients across their life course. To date, no scaled mechanisms to drive pooled procurement or innovation for access to noncommunicable disease medicines have been implemented, even in low-income countries. In addition, in contrast to data on infectious diseases, less routine data on the noncommunicable disease burden and treatment in low-income countries exist, making the market uncertain and fragmented. 

As countries become increasingly responsible for financing their own health systems, value for money, as well as effectiveness and appropriateness of any new technology should be assessed by the government, against the current standard of care relative to investments in other disease areas and health priorities.^11^ Without donor subsidies for specific products, countries may be less likely to pay for certain medicines, even if the products are highly cost–effective. As disease burden shifts, and countries that move away from aid assume more financial responsibility for medicines and tools, governments and patients are trading off constrained resources between health products, and in some cases between health and non-health priorities. Therefore, relative cost–benefit within and across specific disease areas becomes increasingly important. Finally, continued investment is required for radically innovative development and manufacturing processes to lower the absolute costs and to increase product efficacy.

## Moving forward

The expected increase in global poverty as a result of the COVID-19 response^3^ will test the impact and usefulness of existing tools, including for COVID-19 drugs, diagnostics and vaccines. 

While there is a long list of access tools that have been used (Box 1), there has been no formal evaluation or stock taking on what works, why and for which disease types. Multiple tools are often employed together, but they are rarely evaluated to understand what combination works best and in which circumstance. Moving forward, including for the recently launched Access to COVID-19 Tools Accelerator, we suggest a more purposeful approach, based on evidence and learning, and analysis of the differences between what is required for diseases of poverty and global diseases.

We also observed that, with few exceptions, many access tools were designed and implemented by donors, with relatively little engagement from patients or interaction with the systems into which the product is or will be introduced. This weak linkage between the demand side and the supply side will become more of a problem as countries are increasingly becoming the payers. Product developers will need to systematically engage end-users early and often along the product’s development lifecycle, adapting products, as needed, to improve medication adherence, compliance and affordability.
